# Rapamycin Improves Recognition Memory and Normalizes Amino-Acids and Amines Levels in the Hippocampal Dentate Gyrus in Adult Rats Exposed to Ethanol during the Neonatal Period

**DOI:** 10.3390/biom11030362

**Published:** 2021-02-27

**Authors:** Malgorzata Lopatynska-Mazurek, Anna Pankowska, Ewa Gibula-Tarlowska, Radoslaw Pietura, Jolanta H. Kotlinska

**Affiliations:** 1 Department of Pharmacology and Pharmacodynamics, Medical University, Lublin, 20-059 Lublin, Poland; gosia.lopatynska@gmail.com (M.L.-M.); ewa.gibula@umlub.pl (E.G.-T.); 2 Department of Radiography, Medical University, Lublin, 20-081 Lublin, Poland; anna.pankowska@umlub.pl (A.P.); radoslaw.pietura@umlub.pl (R.P.)

**Keywords:** neonatal ethanol, rapamycin, dentate gyrus, amino acids, behavior

## Abstract

The mammalian target of rapamycin (mTOR), a serine/ threonine kinase, is implicated in synaptic plasticity by controlling protein synthesis. Research suggests that ethanol exposure during pregnancy alters the mTOR signaling pathway in the fetal hippocampus. Thus, we investigated the influence of pre-treatment with rapamycin, an mTORC1 inhibitor, on the development of recognition memory deficits in adult rats that were neonatally exposed to ethanol. In the study, male and female rat pups received ethanol (5 g/kg/day) by intragastric intubation at postanatal day (PND 4-9), an equivalent to the third trimester of human pregnancy. Rapamycin (3 and 10 mg/kg) was given intraperitoneally before every ethanol administration. Short- and long-term recognition memory was assessed in the novel object recognition (NOR) task in adult (PND 59/60) rats. Locomotor activity and anxiety-like behavior were also evaluated to exclude the influence of such behavior on the outcome of the memory task. Moreover, the effects of rapamycin pre-treatment during neonatal ethanol exposure on the content of amino-acids and amines essential for the proper development of cognitive function in the dentate gyrus (DG) of the hippocampus was evaluated using proton magnetic resonance spectroscopy (1H MRS) in male adult (PND 60) rats. Our results show the deleterious effect of ethanol given to neonatal rats on long-term recognition memory in adults. The effect was more pronounced in male rather than female rats. Rapamycin reversed this ethanol-induced memory impairment and normalized the levels of amino acids and amines in the DG. This suggests the involvement of mTORC1 in the deleterious effect of ethanol on the developing brain.

## 1. Introduction

Ethanol is a neurotoxic compound and its abuse can cause neural damage, protein oxidation and even neurodegeneration [1]. Gestational alcohol exposure leads to a range of neurodevelopmental disorders, collectively known as Fetal Alcohol Spectrum Disorders (FASD) [2,3]. Alcohol differentially affects a variety of brain regions and neuronal populations [4]; specifically, structural anomalies have been observed in highly vulnerable regions, including the hippocampus, cerebellum and cortex [5,6]. Heavy prenatal alcohol exposure can result in physical, intellectual and/or behavioral impairments [7], which are observed in childhood and last long into adulthood [3]. Binge drinking during the third trimester is particularly harmful to fetal development because in this period higher-order cognition, including executive functions, learning and memory [3,8,9,10,11,12] are developed. 

The dentate gyrus (DG) is a region within the hippocampus that receives its major input from the entorhinal cortex [13,14], and projects to area CA3 of the hippocampus. Previous experiments showed that the DG-specific knockdown of adult neurogenesis impairs spatial and object recognition memory in adult rats [15]. Recent data support the growing evidence for the role of the DG of the hippocampus in novel object recognition (NOR) and especially in novelty detection (critical for pattern separation) [16,17]. Animal work suggests that the CA3 and DG enable accurate object recognition and pattern separation, respectively [17,18]. 

The NOR is a commonly used test to evaluate recognition memory in rodents. In this test, subjects must spontaneously explore a pair of identical objects and, after a delay, distinguish between the now familiar objects and novel objects [19]. This test allows researchers to evaluate short- and long-term memory. In this test, the shorter than 20 min retention interval depends on short-term memory observation, whereas the 240 min interval necessitates an intermediate form of memory [19,20,21]. Long-term memory, at durations of 24 h or longer requires both protein synthesis and gene transcription [22,23]. 

The mammalian target of rapamycin (mTOR) is a highly conserved serine/threonine kinase. This enzyme regulates a variety of cellular processes, including cell growth, proliferation and survival via two distinct multiprotein complexes, mTORC1 and mTORC2. While mTORC1 functions as a nutrient/energy/redox sensor and controls protein homeostasis, mTORC2, through activation of Akt/protein kinase B (PKB) and phosphorylation of Ser473 [24,25], regulates cell survival, proliferation and metabolism [26]. mTORC1 has been implicated in controlling soma and dendritic morphology [25], synaptic plasticity, learning and memory by controlling protein translation [27,28,29,30,31]. 

Published data show that acute inhibition of mTOR has generally been associated with defects in long-term plasticity required for memory [32]; however, inhibition of mTOR can also block the opposite process, the long-term reduction in synaptic responsiveness or long-term depression (LTD) [33]. Preclinical data indicate that the Food and Drug Administration-approved inhibitor of the mTORC1, rapamycin, decreases many effects of ethanol dependence, such as expression of alcohol-induced locomotor sensitization, place preference, as well as excessive alcohol intake and seeking without influence on natural reward [34]. Furthermore, other results show that chronic binge alcohol exposure during pregnancy alters the mTOR(C1) system in the rat fetal hippocampus [35,36]. 

The primary concern of our experiments was to evaluate the protective role of pre-treatment of rapamycin, the mTORC1 inhibitor, during neonatal exposure to ethanol on recognition memory deficits in adult rats. To perform such study, the rat pups (male and female) were exposed to ethanol (5 g/kg/day, intragastrically) during postnatal day (PND) 4-9. Next, rapamycin was given before every ethanol administration during the PND 4-9 period. As adults (PND 59/60), the rats (male and female) were subjected to the NOR task for assessing recognition memory (taking into account the brain development of the rodent, PND 60+ is equivalent to an age of 20 years or more in humans [37] [Semple et al., 2013]). In separate groups of animals, the influence of rapamycin pretreatment before every ethanol administration during PND 4-9 on biomarkers that are currently in use for the diagnosis of FASD in the DG in adult (PND 60) male rats was assess by proton magnetic resonance spectroscopy (^1^HMRS). We evaluated such FASD biomarkers as glutamine, glutamate, taurine and choline that are crucial for proper neurodevelopment [38]. These biomarkers act as neurotransmitters or neurotransmitter precursors in the various brain structures and could take part in FASD-related memory impairment.

## 2. Materials and Methods

### 2.1. Animals

The experiments were carried out according to the National Institute of Health Guidelines for the Care and Use of Laboratory Animals, as well as to the European Community Council Directive for Care and Use of Laboratory Animals (86/609/EEC) and was approved by the Local Ethics Committee (44/2018). Wistar rats used in the study were bred and housed in the vivarium of the Medical University of Lublin, Poland. The vivarium was maintained on a 12 h/12 h light/dark cycle, with lights on at 8:00 AM, and at a controlled temperature of 22 ± 1 °C and humidity of 55 ± 10%. Rats have access to food and water *ad libitum*. For breeding, one male and one female rat were housed together for one week. Beginning 3 weeks after mating, the females was checked each morning and evening for parturition. Following parturition, on PND 3, pups were assigned to experimental groups retaining equal numbers of males and females. These rats were housed with dams to PND 21 then same-sex housed with 2–3 littermates through PND 59/60. Behavioral tests were initiated at PND 59/60 and conducted between 8:00 a.m. and 4:00 p.m.

### 2.2. Drugs and Neonatal Treatment 

On PND 3, male and female rat pups were paw-marked for identification and were assigned into 1 of 6 treatment groups that were used in the behavioral experiments: sham-intubated + 0.9%NaCl (male/female, *n* = 8), sham-intubated + rapamycin (3 mg/kg) (male/female, *n* = 8), sham-intubated + rapamycin (10 mg/kg) (male/female, *n* = 8), ethanol+ 0.9%NaCl (male/female, *n* = 8), ethanol + rapamycin (3 mg/kg) (male/female, *n* = 8), ethanol + rapamycin (10 mg/kg) (male/female, *n* = 8). Additionally, a separate group of 24 rat pups that did not undergo behavioral tests was used in the neurochemical experiments (PND 60): sham-intubated + 0.9%NaCl (*n* = 6), sham-intubated + rapamycin (10 mg/kg) (*n* = 6), ethanol + 0.9%NaCl (*n* = 6), ethanol+ rapamycin (10 mg/kg) (*n* = 6) (see paragraph 2.6). Ethanol (95% *w*/*v*, Polmos, Poznan, Poland) was given on PND 4-9 according to the method described by Goodfellow et al. [39] and MacIlvaneet al. [40] (as a “3rd trimester exposure” model). Pups received ethanol at the dose of 5.0 g/kg/day, 22, 66% *v*/*v*, delivered via intragastric intubation (i.g.) in milk (Bebilon 1 Pronutra Plus) solution. This dose of ethanol produces significant neurotoxicity during the third trimester equivalent and may lead to neurobehavioral deficits [41]. Rapamycin (Selleckchem, Munich, Germany) was dissolved in 0.9% NaCl and given intraperitoneally (i.p.), 1 h before ethanol intubation at the dose of 3 or 10 mg/kg. The rapamycin doses were chosen based on a previous study by Lin et al. [42]. Sham-intubated control rats received identical treatment over PND4-9 but did not receive the ethanol-milk or milk solution.

### 2.3. Procedures

#### 2.3.1. Novel Object Recognition Test

**Figure 1:**
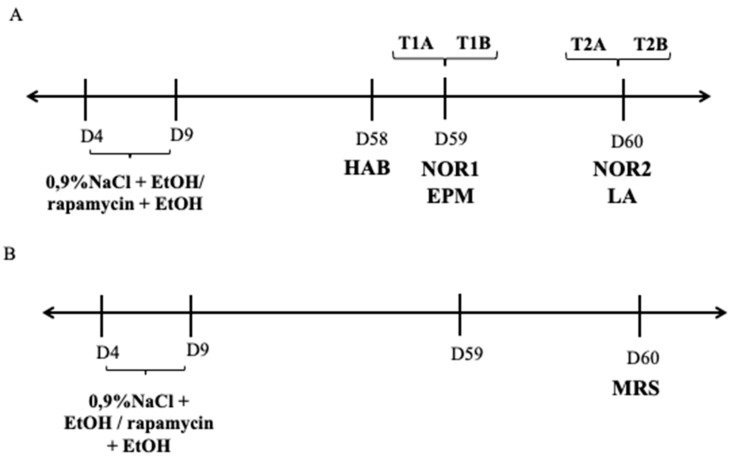
Diagram of experimental design. (**A**) 0.9%NaCl (i.p.); EtOH—ethanol (i.g.); rapamycin (i.p.); HAB—habituation; NOR - novel object recognition; EPM—elevated plus maze; LA—locomotor activity; (**B**) MRS—magnetic resonance spectroscopy; D—day.

The behavioral test was conducted in adult (PND 59) rats (male and female), according to the method described by Marszalek-Grabska et al. [43,44]. The NOR test consisted of three phases—habituation and familiarization phases and test phase. On the first day of study (habituation phase), the rats were placed into an empty plexiglass box (63 cm long × 44.5 cm high × 44 cm wide) for 5 min and could roam freely to form a recognition of the cage and become familiar with the new environment. Next, the test sessions for examining object memory were given. The training (T1A) and test (T1B and T2B) sessions were based on two 3-min sessions. The first testing session (T1B) was separated by a 5 min delay (5 min retention interval, NOR 1) and the second was separated by a 24 delay after the initial training session. During the training, rats explored two identical objects (A1, A2). After 5 min, when the short-term memory of the rats was evaluated, the same rats were subjected to the T1B test with one familiar and one novel object (A2, B) for 3 min. In turn, the T2B session (NOR 2), performed 24 h after the T1B session, evaluated long-term memory. Here, one of the objects was replaced with a new object (A2, C) and the rats were reintroduced and allowed to explore for 3-min. Exploration of the objects is defined as directing the nose toward objects at a distance no more than 1cm or touching and sniffing the objects with the nose. Turning around or sitting on objects is not considered as exploratory behavior [45]. All objects and the box were cleaned with 10% ethanol between each session to avoid the presence olfactory trials. Memory was evaluated by way of the discrimination index (DI). This is the ability to discriminate the novel from the familiar object. DI is calculated by the following formula: (novel object (s)/novel object (s) + familiar object (s) × 100%). An index higher than 50% indicates novel object preference, lower than 50%—familiar object preference and 50%—no preference [46]. The diagram of experimental designs is shown in Figure 1A. 

#### 2.3.2. Elevated Plus Maze (EPM)

The test was performed according to the method described in [47]. The wooden Elevated Plus Maze (EPM) apparatus consisted of two open and two closed arms (50 × 10 cm^2^) arranged in the shape of a plus sign. The closed arms were surrounded by side walls 40 cm high. The maze was elevated 50 cm above the floor. The experiment was conducted in a quiet, dark room lit by 120 lx light. It was started by placing a rat in the central square of the plus maze and allowing it to freely explore the maze for 5 min. The “arms entry” was recorded when all four paws were placed in the arm. The parameters established for each rat was: (a) the time spent in the open arms as a percent of the total time spent exploring both the open and closed arms (Percent Time), and (b) the number of entries into the open arms as a percentage of the total number of entries into both open and closed arms (Percent Entries). The results were scored by video tracking software (Ugo Basile, Italy). The anxiety-like behavior in adult rats (PND 59) exposed to ethanol during PND 4-9 in the EPM test was measured for 5-min. The apparatus was thoroughly cleaned with water after each session to reduce aversive olfactory stimuli. The EPM test was conducted on PND 60, after completing the NOR 1 test in accordance with the schedule previously described [43,48]. 

#### 2.3.3. Locomotor Activity

To exclude the impact of locomotor on the NOR results, the locomotor activity of individual rats was recorded using a photocell apparatus (Porfex, Bialystok, Poland). The animals (PND 60) were placed in an apparatus (a square cage, 60 × 60 cm^2^, made of transparent plexiglass) for measuring locomotor activity (Porfex, Białystok, Poland). The cages were located in a sound-attenuated experimental room with constant lighting (40 W). After 10 min of acclimatization, the total horizontal activity (distance traveled in meters) of individual animals was measured (15 min) by two rows of infrared light-sensitivity photocells placed 45 and 100 mm above the floor. Such method was used in our previous study [43]. The apparatus was thoroughly clean with water between each animal to remove odor traces. The locomotor activity test was conducted after completing the NOR 2 test.

### 2.4. Spectral Analysis and Quantification of Neurochemicals in the Dentate Gyrus of the Hippocampus In Vivo

Proton magnetic resonance spectroscopy(^1^HMRS) experiments were performed on a 7T MRI scanner (70/16 Pharma Scan, Bruker Biospin, GmbH, Germany), using a 72 mm transceiver RF coil and a 10 mm receive-only surface loop coil. The animals were anesthetized with isoflurane/oxygen mixture (3.5% for induction and 1.7–2.5% for maintenance). Rats breathed freely during the ^1^HMRS studies and the anesthetic concentration was adjusted to maintain the respiratory rate ~50 bpm. Body temperature was maintained at 37 °C using circulating water. Both respiratory rate and body temperature were continuously monitored with an MR-compatible Small Animal Monitoring System (SA Instruments, Inc., New York, NY, USA). MRS protocol for each rat lasted approximately 60 min. First, a three-slice (axial, midsagittal, and coronal) scout using fast low-angle shot MRI was obtained to localize the rat brain and enable voxel positioning for MRS. 

Point resolved spectroscopy sequences (PRESS) with the following parameters: bandwidth 3 kHz, 4096 complex data points, TR 2.500ms, TE 16 ms, 1024 averages were used for data acquisition. Field homogeneity of the volume of interest (VOI) was achieved via Localized Shim procedure (Bruker BioSpin spec, Ettlingen, Germany), the full width at half maximum (FWHM) was typically in the range of 7 to 13 Hz. The water signal was suppressed using eight variable power RF pulses with optimized relaxation delays (VAPOR). Additional non-water suppressed spectra were acquired to allow for the normalization of neuro metabolite concentrations to the concentration of in vivo brain water. 

**Figure 2:**
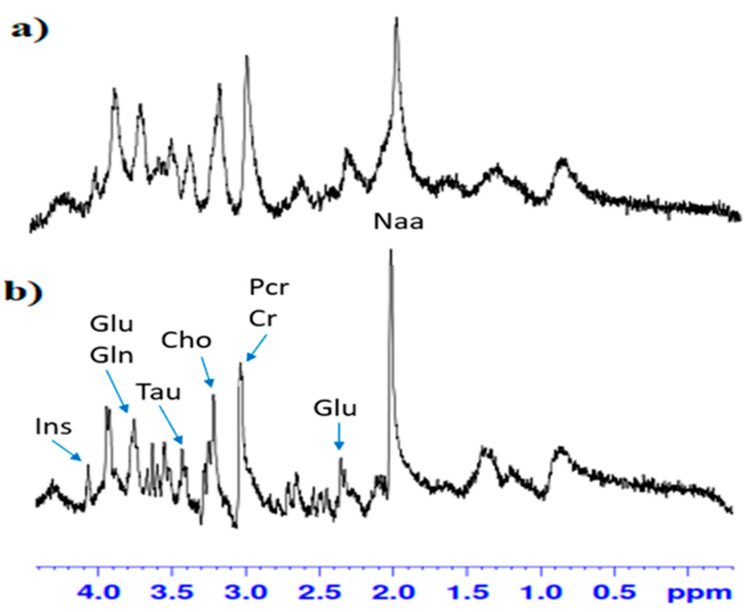
Typical in-vitro proton magnetic resonance spectroscopy (^1^HMRS) spectrum in the dentate gyrus (DG) of hippocampus (**a**) in group fed with alcohol, (**b**) in control group. 7T Bruker, animal system. Inositol (Ins), glutamate (Glu), glutamine (Gln), taurine (Tau), choline (Cho), total creatine (PCr + Cr), N-acetylaspartate (Naa).

MR spectra were processed using LC Model (Linear Combination of Model Spectra) software (Version 6.3-1). Window of spectra analysis in the LC Model package were from 0.2 to 4.6 ppm. Fitting spectra through the LC Model package allowed for reliable identification and quantification of metabolites, which was reflected in their CRLB (Cramer Rao Lower Bounds) designation and determined the lower limit of statistical error of the fitting accuracy for the line of individual metabolites. This was calculated by LC Model software, the manual of which states that only metabolites with CRLB estimation below 20% can be considered as reliably quantified. Fitting the spectra with the use of LC Model allowed for the correct designation of 9 metabolic profiles with the CRLB below 20%: N-acetylaspartate-2% (Asp), glutamate-2% (Glu), glutamine-6% (Gln), myo-inositol-3% (Ins), phosphorylcholine-3% (PCho), taurine-3% (Tau), creatine-6% (Cr), phosphocreatine-5% (PCr), γ-aminobutyric acid-11% (GABA). An illustrative example of the MR image with the MRS voxel positioned in the dentate gyrus (DG) of the hippocampus is presented in Figure 2.

Proton MRS spectra were acquired for each animal over the 2.0 × 2.0 × 5.5 mm^3^ VOI placed at the right hippocampus. On PND 60, the effect of rapamycin pre-treatment before every ethanol administration during PND 4-9 on the content of the amino acids and amines that are considered important for proper memory process development in the DG of the hippocampus was evaluated using ^1^HMRS. The diagram of experimental designs was shown in Figure 1B. 

### 2.5. Statistical Analyses

The results were analyzed using the GraphPad Prism version 8.00 for Windows, Graph-Pad Software (San Diego, CA, USA). The statistical significance of drug effects from the ^1^HMRS experiment, as well as the behavioral and biochemical tests were analyzed by two- or three-way analysis of variance (ANOVA) with repeated measures. This was followed by Bonferroni’s and Tukey’s post-hoc test. The results were presented as means ± standard errors of means (SEM) of values. A *p* value less than 0.5 was considered statistically significant for all tests.

## 3. Results

### 3.1. The Influence of Rapamycin Pre-Treatment before Every Ethanol Administration during PND 4-9 on the Short-and Long-Term Memory in the NOR Test in Adult (PND 59/60) Male and Female Rats

In the short-term memory recognition test given 5-min (T2B) after training, three-way analysis of variance (ANOVA) did not show significant effects of pre-treatment (rapamycin effect) [F(2,84) = 2.219; *p* > 0.05], treatment (ethanol effect) [F(1.84) = 0.7629; *p* > 0.05], sex of rats [F(1,84) = 0.7879; *p* > 0.05], as well as pre-treatment x treatment [F(2,84) = 0.3808; *p* > 0.05], pre-treatment x sex [F(2,84) = 0.1636; *p* > 0.05], treatment x sex [F(1,84) = 0.1579; *p* > 0.05] and pre-treatment x treatment x sex [F(2,84) = 2.045; *p* > 0.05] interactions. The post-hoc test also did not reveal statistically significant differences between groups (*p* > 0.05).

**Figure 3:**
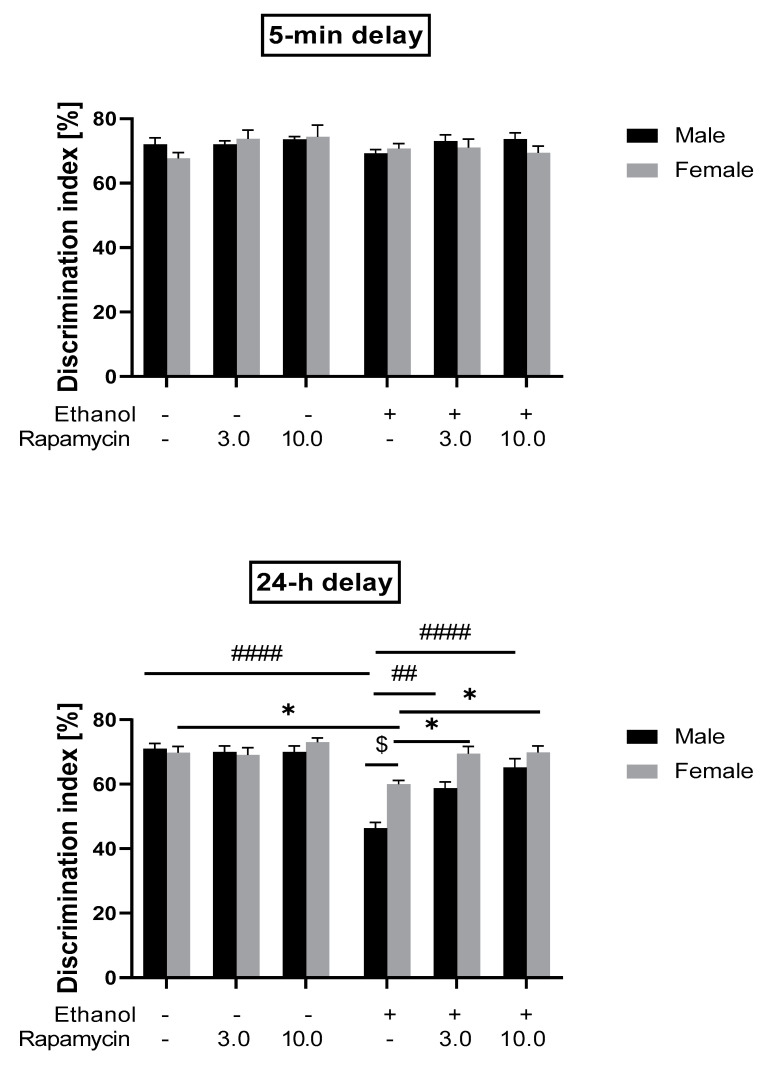
The influence of rapamycin pre-treatment (3 and 10 mg/kg, i.p.) during ethanol administration (postnatal day (PND) 4-9) on short- and long-term memory deficits in the novel object recognition (NOR) test (expressed as discrimination index) in adult (PND 59-60) rats. Results are expressed as mean ± SEM of 8 subjects per group. * *p* < 0.05 vs. Control; ^##^ *p* < 0.01, ^####^ *p* < 0.001 vs. EtOH; ^$^ *p* < 0.05 vs. EtOH.

In the long-term memory recognition test that was given 24-h after training (T2C), three-way ANOVA showed the significant effect of pre-treatment (rapamycin effect) [F(2,84) = 16.05; *p* < 0.0001], treatment (ethanol effect) [F(1,84) = 45.87; *p* < 0.0001], sex of rats [F(1,84) = 10.60; *p* < 0.01], pre-treatment x treatment [F(2,84) = 13.26; *p* < 0.0001], treatment (ethanol effect) x sex [F(1,84) = 8.946; *p* < 0.01] and pre-treatment x treatment x sex [F(2,84) = 3.127; *p* < 0.05] interactions. However, three-way ANOVA did not indicate significant pre-treatment x sex interaction [F(2,84) = 0.2950; *p* > 0.05]. Post-hoc test showed that adult male rats exposed to ethanol (5 mg/kg, i.g.) neonatally (PND 4-9) explored the novel object significantly less than the control group (shame-intubated) (*p* < 0.001) and ethanol-treated female rats (*p* < 0.05). In the ethanol-treated male rats, both 3 mg/kg and 10 mg/kg, i.p. of rapamycin given before every ethanol administration during PND 4-9 increased (*p* < 0.01 and *p* < 0.0001, respectively) the DI. Post-hoc testing also showed that male rats pre-treated with rapamycin at the dose of 3 mg/kg and 10 mg/kg, i.p. spent more time exploring the novel object, as compared to ethanol-treated female rats. The results suggest the protective effect of rapamycin on the development of ethanol-induced memory impairment, particularly in male rats (see Figure 3).

### 3.2. The Influence of Rapamycin Pre-Treatment before Every Ethanol Administration during PND4-9 on Anxiety-Like Behavior in Adult Male and Female (PND 59) Rats Using the EPM Test

**Figure 4:**
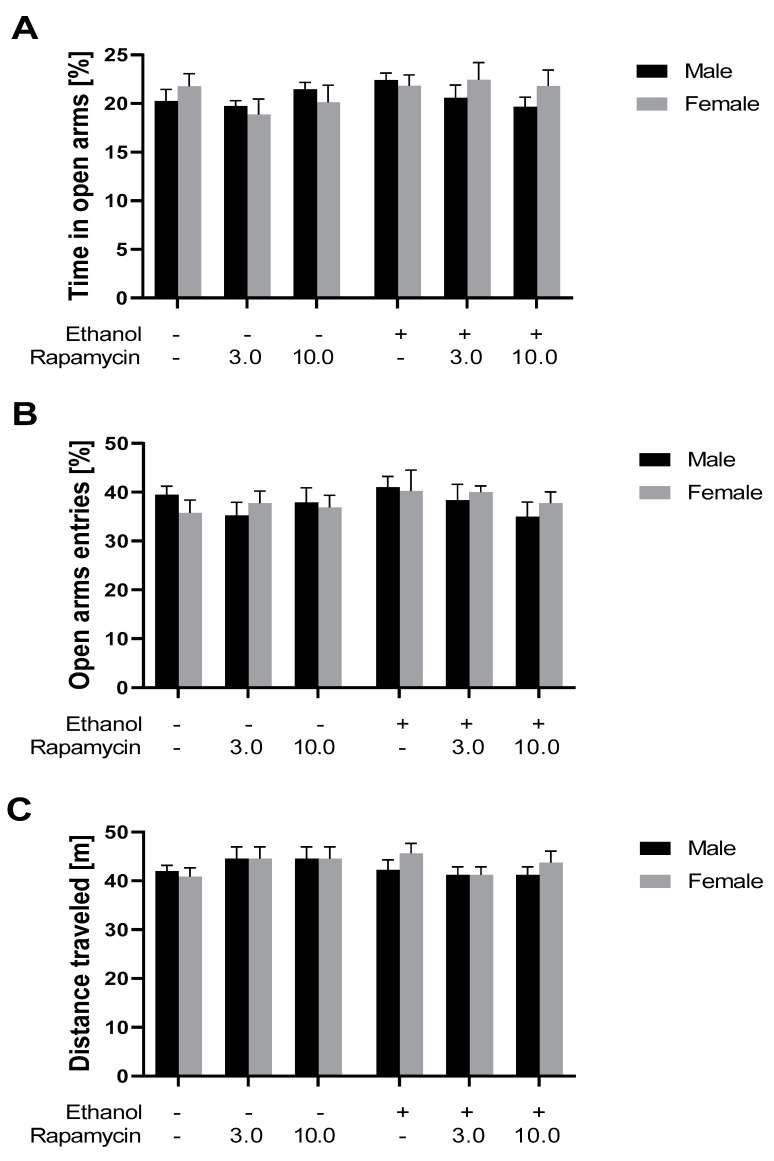
The influence of rapamycin pre-treatment (3 and 10 mg/kg, i.p.) during ethanol administration (PND 4-9) on anxiety-like behavior (PND 59) and locomotor activity (PND 60) in adult male and female rats measured in the elevated plus maze (EPM). (**A**) Percent of time in the open arms of the EPM apparatus; (**B**) percent of open arm entries in the EPM apparatus; (**C**) locomotion measured in the locomotor activity boxes. Results are expressed as mean ± S.E.M. of 8 subjects per group.

In adult rats, three-way ANOVA did not reveal the statistically significant effect of rapamycin pre-treatment [F(2,84) = 0.8365, *p* > 0.05], ethanol treatment [F(1,84) = 2.136, *p* > 0.05], sex of rats [F(1,84) = 0.3529, *p* > 0.05] and interactions between these factors on the percent of time spent by male adult rats in the open arms of the EPM apparatus (see Figure 4A). 

Furthermore, three-way ANOVA did not show the statistically significant effect of rapamycin pre-treatment [F(2,84) = 0.6924; *p* > 0.05], ethanol treatment [F(1,84) = 0.9953, *p* > 0.05], sex of rats [F(1,84) = 0.02141, *p* > 0.05] and interactions between factors—on the percent of entries into the open arms of EPM apparatus in adult male rats (see Figure 4B).

### 3.3. The Influence of Rapamycin Pre-Treatment before Every Ethanol Administration during PND 4-9 on Locomotor Activity in adult (PND 60) Male and Female Rats 

Statistical analysis of the locomotor activity of male rats (three-way ANOVA) did not show the significant effect of rapamycin pre-treatment [F(2,84) = 0.1932, *p* > 0.05], ethanol treatment [F(1,84) = 0.7251, *p* > 0.05], sex of rats [F(1,84) = 0.4677, *p* > 0.05] and interactions between factors (see Figure 4C).

### 3.4. The Influence of Rapamycin Pre-Treatment before Every Ethanol Administration during PND 4-9 on Changes in Glutamate, Glutamine Concentration and Glutamate/Glutamine Ratio in the Hippocampal DG Measured by MRS Experiment in Adult (PND 60) Male Rats

#### 3.4.1. Glutamate

During the MRS experiment in male rats (PND 60), two-way ANOVA indicated a statistically significant effect of rapamycin pre-treatment [F(1,20) = 7.370; *p* < 0.05], ethanol treatment (F(1,20) = 6.191; *p* < 0.05) and interaction between these two factors [F(1,20) = 10.03; *p* < 0.01] on glutamate (Glu) levels in the DG. Post-hoc analysis (Tuckey’s test) showed that ethanol administrated for 5 days (PND 4-9) at the dose 5 mg/kg, i.g. increased levels of Glu in the DG, as compared to the control group (*p* < 0.01). Moreover, pre-treatment of rapamycin at the dose 10 mg/kg significantly reduced the elevated Glu level in adult male rats (*p* < 0.01) (Table 1). The effect was comparable to the control group. 

#### 3.4.2. Glutamine

During the MRS experiment in male rats (PND 60), two-way ANOVA showed that the statistical effect of rapamycin pre-treatment was almost significant at 95% confidence level and significant at 90% confidence level [F(1,20) = 3.47; *p* = 0.077]. The significant effect of ethanol treatment [F(1,20) = 4.57; *p* < 0.05] and significant interactions between this two factors [F(1,20) = 6.30; *p* < 0.05] in glutamine (Gln) level in the DG were also noted. Post-hoc analysis (Tuckey’s test) showed that ethanol administrated for 5 days (PND 4-9) at the dose of 5 mg/kg, i.g. increased levels of Gln in the DG (*p* < 0.05), as compared to the control group. In contrast, pre-treatment with rapamycin at the dose 10 mg/kg attenuate the elevated Gln level in adult male rats (*p* < 0.05) (see Table 1). The effect was comparable to the control group. 

### 3.5. The Influence of Rapamycin Pre-Treatment before Every Ethanol Administration during PND 4-9 on Gamma-Aminobutyric Acid, Taurine and Total Choline Concentration in the Hippocampal DG Measured by MRS Experiment In Adult (PND 60) Male Rats 

#### 3.5.1. Taurine

Two-way analysis of variance (ANOVA) with repeated measures revealed a significant effect of rapamycin pre-treatment [F(1,20) = 10.93, *p* < 0.01], ethanol treatment [F(1,20) = 32.54, *p* < 0.0001] and interactions between these two factors [F(1,20) = 12.22, *p* < 0.01] in taurine (Tau) concentration [mmol/l] in the DG. Post-hoc analysis (Bonferroni’s multiple comparisons test) showed that ethanol administrated for 5 days (PND 4-9) at the dose 5 mg/kg decreased levels of Tau concentrations [mmol/l] in the DG of adult male rats, as compared to the control group (*p* < 0.0001). Moreover, pre-treatment of rapamycin at the dose of 10 mg/kg, i.p. significantly increased Tau level, compared to the ethanol group, in the DG of adult male rats (*p* < 0.0001) (see Table 1). The effect was comparable to the control group. 

#### 3.5.2. Total Choline

Two-way analysis of variance (ANOVA) with repeated measures showed a significant effect of rapamycin pre-treatment [F(1,20) = 5.467, *p* < 0.05], ethanol treatment [F(1,20) = 8.184, *p* < 0.01)] and interactions between these two factors [F(1,20) = 7.563, *p* < 0.05].

Post-hoc analysis (Bonferroni’s multiple comparisons test) showed that ethanol administrated for 5 days (PND 4-9) at the dose 5 mg/kg decreased levels of Cho concentrations [mmol/l] in the DG of adult male rats, as compared to the control group (*p* < 0.01). In addition, pre-treatment of rapamycin at the dose of 10 mg/kg, i.p. significantly increased Tau level compared to the ethanol group in the DG of adult male rats (*p* < 0.01) (see Table 1). The effect was comparable to the control group. 

### 3.6. The Influence of Rapamycin Pre-Treatment before Every Ethanol Administration during PND 4-9 on N-acetylaspartate, Inosytol and Creatine Concentration in the Hippocampal DG Measured by MRS Experiment in Adult (PND 60), Male Rats

#### 3.6.1. N-Acetylaspartate

Two-way analysis of variance (ANOVA) with repeated measures did not reveal a significant effect of rapamycin pre-treatment [F(1,20) = 0.3839, *p* > 0.05], ethanol treatment [F(1,20) = 0.8605, *p* > 0.05] and pre-treatment x treatment interactions [F(1,20) = 1.627, *p* > 0.05)] on N-acetylaspartate (Naa) levels in the DG of male adult rats (PND 60). Post-hoc analysis (Tuckey’s test) did not show significant differences between groups (*p* > 0.05) (see Table 1). 

#### 3.6.2. Inosytol

Two-way analysis of variance (ANOVA) with repeated measures did not reveal a significant effect of rapamycin pre-treatment [F(1,20)= 0.0001472, *p* > 0.05] or of ethanol treatment [F(1,20)= 0.7830, *p* > 0.05)], but showed statistically pre-treatment x treatment interactions (F(1,20) = 8.837, *p* < 0.01) on inosytol (Ins) levels in the DG of adult male rats (PND 60). Post-hoc analysis (Tuckey’s test) did not show significant differences between groups (*p* > 0.05) (see Table 1). 

#### 3.6.3. Total Creatine

Two-way analysis of variance (ANOVA) with repeated measures did not indicate the significant effect of rapamycin pre-treatment [F(1,20) = 1.376, *p* > 0.05], pre-treatment x treatment interactions [F(1,20) = 1.020, *p* > 0.05], but showed a statistically significant effect of ethanol treatment (F(1,20) = 4.473, *p* < 0.05) on creatine (Cr) levels in the DG of adult male rats (PND 60). Post-hoc analysis (Tuckey’s test) did not show significant differences between groups (*p* > 0.05) (see Table 1).

## 4. Discussion

The results of this study suggest that the brain mechanisms underplaying learning and memory in recognizing new objects are vulnerable to the effect of neonatal ethanol exposure (PND 4-9). The main experimental finding indicated that long-term (24 h) recognition memory is selectively impaired in adult rats in the NOR test. The deficits were much more pronounced in male than female rats and were accompanied by the disruption of amino acids and amines concentrations (mainly Glu, Tau and also Gln and Cho) in the DG. The administration of the mTORC1 inhibitor, rapamycin, before every ethanol exposure during PND 4-9, ameliorated long-term memory impairment induced by ethanol and normalized the concentration of Glu, Gln or Tau. 

### 4.1. Rapamycin Prevents Ethanol-Induced Recognition Memory Impairment 

In humans, alcohol consumption during pregnancy leads to FASD with deleterious effects on recognition memory after birth [3,49,50]. Studies in animals provide, however, mixed results. Thus, intact recognition performance has been demonstrated during adulthood following neonatal/prenatal ethanol exposure, with a 30 min [51] and 5 min delay [52] between the sample and testing phase. Other studies showed that recognition memory was impaired only after a 15 min delay in adult mice exposed to ethanol on gestational day 8 [53] or 20 min in adult rats exposed to binge-like doses of ethanol during early postnatal life [46]. Our results show, similar to Wilson et al. [54], that neonatal alcohol exposure impacts recognition memory after a longer (e.g., 24 h) delay between the sample and testing phases. This effect was significant in male and female rats, although more pronounced in the males. Additionally, anxiety-like behavior and changes in locomotor activity were not observed in these animals. Thus, our data suggests that neonatal ethanol administration did not have significant sensory, motor or emotional influence on the recognition memory outcome. In humans, the diagnosis of FASD is sex- and age-dependent, with males being more likely to be diagnosed with FASD at earlier ages, perhaps because females only meet certain diagnostic criteria at later ages or because the characteristics of FASD are more pronounced in males than in females [55]. Interestingly, sex-differences have also been identified in animal recognition memory tasks [56] and our current results confirm this. It should be mentioned that there are also data suggesting no observable sex differences in outcome as a function of neonatal ethanol exposure in animals [57]. Thus, our data can confirm sex-differences presented in humans with FASD [55] and suggests that deficits in recognition memory are probably a consequence of reductions in neural activation during memory consolidation and retrieval—two key elements of recognition memory that appear to be targeted by prenatal alcohol [53]. 

The hippocampus is engaged in the consolidation of non-spatial object recognition memory [58]. Ethanol exposure over postnatal days PND 4-9 in rodents, a period comparable to the human third trimester [59,60], impairs hippocampal neurodevelopment and produces significant reductions in dendritic spine density, neurogenesis and long-term potentiation (LTP) [61,62,63,64]. Rats neonatally exposed to ethanol showed impaired DGcells because neurogenesis in this subregion occurs postnatally in rats [64,65,66]. 

Published data indicate that hippocampal lesions result in impaired object recognition memory in humans and primates [67,68,69,70]. Furthermore, Clark et al. [71] found that rats with pre-training hippocampal lesions exhibited impaired object recognition memory with long retention intervals imposed (>15 min), but not short retention intervals (<15 min). 

Taking into account these data, we can anticipate that impaired hippocampus and (particularly) the DG are responsible for long-term recognition memory deficits in our study. Recent studies showed dynamic bidirectional changes in long term potentiation (LTP) and long term depression (LTD) in the DG following prenatal ethanol exposure in rats [72,73]. What is more, male LTP and LTD are reduced in magnitude, while in female only LTP was affected [72]. Other studies in rats suggest that neonatal ethanol exposure induces long-term alternation of synaptic plasticity involving N-methyl-D-aspartate (NMDA) receptors, leading to an aberrant LTD that may also participate in long-lasting cognitive deficits in FASD [74]. In our study, the most significant effects were seen in male rats. We can, therefore, conclude that attenuations of both LTP and LTD expression are involved in recognition memory impairment induced by neonatal ethanol treatment. Herein, rapamycin pre-treated rats before every ethanol administration during PND 4-9 indicated intact recognition memory, suggesting that rapamycin has a protective role. Because rapamycin is an mTORC1 inhibitor [75], our data suggest the involvement of mTORC1 in cognitive impairment induced by neonatal alcohol exposure. 

### 4.2. Rapamycin Prevents Neonatal Ethanol Induced Developmental Alteration in the DG. MRS Experiments

Glutamine is the most significant precursor for the major excitatory neurotransmitter, glutamate [76,77]. Our study of adult rats shows an increase in glutamate and glutamine level in the DG in animals that received ethanol during PND 4-9. Thus, our study extended the previously published notion that an increase in glutamate and glutamine in the hippocampus was observed in alcohol-exposed offspring [78]. 

An increase in glutamate in the developing brain was noted in response to traumatic brain injury [79], hypoxia-ischemia [80] or maternal stress [81]. In the developing brain, acute concentration of glutamate leads to acute neurotoxicity, as well as impairment in neurotransmitter programming, receptor expression, neuronal migration and synapse maturation, and has been linked with numerous long-term behavioral deficits [82]. Indeed, animal studies showed that initial ethanol exposure induces the hyperglutamatergic state [83,84], which triggers apoptotic neurodegeneration [85,86]. Glutamate action at N-methyl-D-aspartate (NMDA) receptors in the hippocampus is necessary to produce LTP in the hippocampus [87], as well as object recognition memory with long retention interval [88,89]. Our data indicate that neonatal ethanol exposure induces significant increase in glutamate that may be responsible for the neuronal damage observed in the DG following alcohol exposure and recognition memory impairment in adult rats. 

In turn, choline is a precursor of the acetylcholine neurotransmitter that is implicated in learning and memory. Choline affects and alters the hippocampal cholinergic system that is essential for memory functioning [90]. Rodent data demonstrate that the hippocampus and memory processes are targets of prenatal alcohol exposure (PAE) and that supplementation with choline can reduce the severity of these induced learning and memory deficits [91]. Recently published data show that the reduction of cholinergic tone impairs consolidation of object recognition memory measured at 24 h [92]. Our study supports this data and indicated deficits in choline levels in the DG of adult rats that neonatally received alcohol. Hence, rapamycin pre-treatment before every ethanol administration (PND 4-9) reversed this ethanol effect. 

Taurine is an amino acid essential during development and has been found to be protective against neurotoxicity and various tissue damages, including those from alcohol exposure. Taurine acts as a trophic factor during central nervous system (CNS) development, in structural integrity of cell membranes and in regulating calcium homeostasis. Moreover, it is an antioxidant, an osmolyte, a neuroprotective and a neuromodulating agent. Previous studies indicated that prenatal alcohol exposure decrease taurine level in the fetal brain, especially in the hippocampus [93]. In our studies, alcohol administration (PND 4-9) decreased taurine level in the DG. Thus, both previous and our studies show that taurine can be a key mechanistic component involved in the neuropathology underlying behavioral deficits of FASD. Rapamycin pre-treatment ameliorated these changes. 

Other biomarkers such as N-acetylaspartate (NAA), creatinine or inositol were not significantly changed in the hippocampal GD in our study. Our results support [94] and partially differ to [95,96] in assessing neurochemistry after perinatal alcohol exposure. However, a direct comparison of the results is difficult given different brain structures, ages of either exposure of testing, as well as of ethanol exposure patterns. 

### 4.3. Potential Mechanism of Rapamycin 

The potential mechanism involved in FASD protection by rapamycin is largely unknown. However, recent analysis of brain proteins in a FASD rat model identified the proteins involved in oxidative stress, mitochondrial dysfunction, and mTOR as major pathways in the cortex and hippocampus directly related to FASD neuropathology [36]. Furthermore, gestational chronic binge alcohol exposure alters mTORC1 signaling, its activity indices and related downstream pathways in the fetal hippocampus [36]. Although the limitation of our study is a lack of experiments concerning the influence of ethanol on the mTORC1 signaling pathway in rats exposed neonatally to ethanol, there are published data showing that rapamycin mitigated ethanol-induced reactive oxygen species (ROS) production and neuronal cell death in SH-SY5Y cells and in the mouse developing brain [86]. In our study, rapamycin pre-treatment prevented the deleterious effects of ethanol on neurochemical profiles in the DG of male rats neonatally exposed to ethanol. The outcome of our work supports previous published data that ethanol modified the mTOR(C1) signaling pathway in the fetal hippocampal DG and cortex [35,36]. Furthermore, rapamycin as a mTORC1 inhibitor protected the DG cells against the changes in concentration of Glu, Gln, Tau and Cho induced by neonatal alcohol exposure. One of the suggested/hypothesized mechanisms for rapamycin induced FASD protection is the induction of autophagy. This is pointed out by other authors [36,97,98]. Indeed, several authors pointed out that the effects of rapamycin are stronger in females than in males [99,100]; however, such an outcome is controversial [101], yet it also confirms our data. 

## 5. Conclusions

Our data demonstrate that neonatal ethanol exposure induced changes in the DG proteins involved in neuronal growth and differentiation, which may directly relate to recognition memory deficits, especially in long-term memory associated with FASD. The changes observed in this study confirm the role of mTORC1 in the neurodegenerative effects induced by neonatal ethanol exposure. Future studies are needed to determine the exact mechanism of rapamycin-induced neuroprotection. Such study may facilitate development of targeted pharmacological therapeutic strategies for an FASD.

## Figures and Tables

**Table 1:** Glu, Gln, Tau, Naa, Ins, Cho, Cr concentrations in the hippocampal DG in adult male rats. Results are expressed as mean ± S.E.M. of 6 subjects per group.

	Control	R10	EtOH	EtOH + R10
Glu	7.423833	7.531667	8.771667 **	7.3698333 ^##^
Gln	2.897333	3.029833	3.8481667 *	2.9535 ^#^
Tau	5.569167	5.584333	5.656333 ^###^	6.386167 ***
Naa	3.661	3.257	3.191333	3.3311667
Cho	1.124667	1.189667	1.1365	1.3233333
Ins	5.357667	5.606667	5.573	5.2195
Cr	6.986333	6.860833	7.175	7.033667

* *p* < 0.05; ** *p* < 0.01; *** *p* < 0.001 vs. Control; ^#^ *p*< 0.05; ^##^ *p* < 0.01; ^###^ *p* < 0.001 vs. EtOH. Control- 0.9% NaCl; R10- rapamycin 10 mg/kg; EtOH- Ethanol.

## Data Availability

The data presented in this study are available on request from the corresponding author.
